# Long non-coding RNA H19 promotes proliferation in hepatocellular carcinoma cells via H19/miR-107/CDK6 axis

**DOI:** 10.32604/or.2023.030395

**Published:** 2023-09-15

**Authors:** ARCHITTAPON NOKKEAW, PANNATHON THAMJAMRASSRI, NAPHAT CHANTARAVISOOT, PISIT TANGKIJVANICH, CHAIYABOOT ARIYACHET

**Affiliations:** 1 Department of Biochemistry, Faculty of Medicine, Chulalongkorn University, Bangkok, 10330, Thailand; 2 Center of Excellence in Hepatitis and Liver Cancer, Faculty of Medicine, Chulalongkorn University, Bangkok, 10330, Thailand; 3 Department of Biochemistry, Medical Biochemistry Program, Faculty of Medicine, Chulalongkorn University, Bangkok, 10330, Thailand; 4 Center of Excellence in Systems Biology, Faculty of Medicine, Chulalongkorn University, Bangkok, 10330, Thailand

**Keywords:** HCC, H19, Long-noncoding RNA, MicroRNA, Cyclin-dependent kinase, CDK6

## Abstract

Hepatocellular carcinoma (HCC) is the leading cause of cancer death worldwide; nevertheless, current therapeutic options are limited or ineffective for many patients. Therefore, elucidation of molecular mechanisms in HCC biology could yield important insights for the intervention of novel therapies. Recently, various studies have reported dysregulation of long non-coding RNAs (lncRNAs) in the initiation and progression of HCC, including H19; however, the biological function of H19 in HCC remains unclear. Here, we show that knockdown of H19 disrupted HCC cell growth, impaired the G1-to-S phase transition, and promoted apoptosis, while overexpression of H19 yielded the opposite results. Screening for expression of cell cycle-related genes revealed a significant downregulation of CDK6 at both RNA and protein levels upon H19 suppression. Bioinformatic analysis of the H19 sequence and the 3′ untranslated region (3′ UTR) of *CDK6* transcripts showed several binding sites for microRNA-107 (miR-107), and the dual luciferase reporter assay confirmed their direct interaction with miR-107. Consistently, blockage of miR-107 activity alleviated the growth suppression phenotypes induced by H19 downregulation, suggesting that H19 serves as a molecular sponge for miR-107 to promote CDK6 expression and cell cycle progression. Together, this study demonstrates a mechanistic function of H19 in driving the proliferation of HCC cells and suggests H19 suppression as a novel approach for HCC treatment.

## Introduction

Hepatocellular carcinoma (HCC) is the most common primary liver malignancy and accounts for more than 75% of liver cancer cases, with the highest incidence in Asian countries [1,2]. HCC develops in patients with risk factors such as viral hepatitis, alcohol addiction, and metabolic-associated fatty liver disease (MAFLD) [3]. Although the mortality rates of HCC have increased in many countries over the past decade, the therapy for HCC remains challenging due to late diagnosis and limited treatment options [4,5]. Thus, improved understanding of the molecular pathogenesis of HCC is required for the discovery of novel diagnostic markers and therapeutic targets.

In recent years, various studies have observed dysregulation of long non-coding RNAs (lncRNAs) in the initiation and progression of HCC [6]. In particular, the lncRNA H19 has been found to be overexpressed in HCC tumors, and patients with high expression of *H19* have a poor prognosis [7–12]. The maternally imprinted lncRNA H19 is strongly expressed during embryogenesis and controls embryonic growth as well as expression of the Imprinted Gene Network [13–15].

However, the biological function of H19 in cancers, including HCC, remains controversial. While most HCC studies support its oncogenic function, a few report the opposite results [16,17]. Therefore, further investigation into the molecular mechanisms of H19 in cellular function would help resolve its complex role and advance our understanding of H19 in the pathophysiology of HCC.

H19 has been shown to be involved in multiple processes in the initiation and progression of HCC, including cell proliferation, apoptosis, invasion, drug resistance, and metastasis [16,17]. Nevertheless, the role of H19 in regulation of the cell cycle remains to be further explored. Sustaining proliferative forces along with insensitivity to antigrowth signals is considered a hallmark of cancer cells [18]. Altered expression of cell cycle-related genes such as cyclin-dependent kinases (CDKs) and their associated cyclins is observed during the transformation of normal cells into malignant cancers [19]. Recent research on lncRNAs shows that many of them are critical for the regulation of cell cycle-related genes via various mechanisms at the transcriptional and post-transcriptional levels [20,21]. Dysregulation of these cell cycle regulatory lncRNAs is associated with various cancers and could promote tumorigenesis [20,21]. Thus, molecular studies of the lncRNA function in the cell cycle might lead to the identification of novel targets for cancer therapy or diagnosis. In this work, we aim to explore the role of the lncRNA H19 in regulating the cell cycle of HCC cells and dissect the molecular mechanisms underlying the oncogenic function of H19.

## Materials and Methods

### Cell culture

Human THLE-2 and HepG2 cell lines were obtained from the American Type Culture Collection (ATCC, Manassas, VA, United States; Cat #CRL-2706 and HB-8065, respectively). JHH-4 and Huh7 cell lines were obtained from the Japanese Collection of Research Bioresources (JCRB) Cell Bank (Osaka, Japan; Cat #JCRB0435 and JCRB0403, respectively). THLE-2 cells were maintained in bronchial epithelial cell-growth medium (Lonza, Basel, Switzerland; Cat #CC-3171) with growth supplements according to the ATCC-recommended protocol. HepG2 and Huh7 cells were cultured in low glucose DMEM (Gibco, Detroit, MI, United States), supplemented with 10% fetal bovine serum (FBS) (Cytiva, Marlborough, MA, United States) and 1X Gibco™ Antibiotic-Antimycotic (Gibco, Detroit, MI, United States) in a humidified atmosphere of 5% CO_2_ at 37°C. The culture medium and conditions for JHH-4 cells were similar to those of HepG2 and Huh7 cells, except for using Eagle’s minimal essential medium (EMEM) as the basal medium.

### Loss-of-function experiments

To inhibit the function of H19, we employed lentivirus-mediated short-hairpin RNA (shRNA) expression that can be temporally controlled by doxycycline (Dox) treatment [22]. Annealed oligonucleotides of H19-specific and control shRNA sequences were cloned into a lentiviral vector, Tet-LKO-Puro (AddGene Plasmid #21915), and confirmed for proper insertion by Sanger sequencing. Lentivirus was produced in HEK293FT cells and used to establish Dox-inducible shRNA cell lines as previously described [23]. To suppress the activity of microRNA-107 (miR-107), we cloned a sequence of antagomiR against miR-107 into Tet-LKO-Puro and established transgenic HCC lines with Dox-inducible expression of antagomiR. All primers used for cloning can be found in Suppl. Table 1.

### Gain-of-function experiments

A plasmid for overexpression of H19 (pcDNa3.1(+)_A009-H19, AddGene Plasmid #122473) or a negative control plasmid was transfected into HCC cells using Lipofectamine® 3000 reagent (Invitrogen, Carlsbad, CA, United States) according to the manufacturer’s protocol. A total of 500 ng of plasmid was used for transfection per well of a 24-well plate. Transfected cells were employed for subsequent analysis after 48 h posttransfection. For overexpression of miR-107, the pre-miRNA sequence of miR-107 was cloned into a lentiviral vector as previously described [23]. Primers used for cloning can be found in Suppl. Table 1.

### MTT, apoptosis, and bromodeoxyuridine (BrdU) assay

Cell proliferation was determined using an MTT-based colorimetric assay (Invitrogen, Carlsbad, CA, United States) according to the manufacturer’s protocol. Briefly, cells were incubated in their basal medium containing 0.5 ug/mL of MTT for 60 min. Then, the insoluble formazan crystals were dissolved in DMSO and quantified by measuring the absorbance at 570 nanometers with a spectrophotometer. Detection of apoptosis was accomplished by the Caspase-Glo® 3/7 assay system (Promega, Madison, WI, United States) and staining with Apopxin Green Indicator (AbCam, Waltham, MA, United States) according to the manufacturers’ protocols. For BrdU incorporation, cells were incubated with culture medium supplemented with 10 µM BrdU (AbCam, Waltham, MA, United States) for 30–60 min before fixation with 4% paraformaldehyde. Cells were permeabilized and denatured with 2 N hydrochloric acid prior to detection by immunofluorescence with an anti-BrdU antibody (1:200 dilution; Cat #sc-32323; Santa Cruz, Dallas, TX, United States), followed by incubation with Alexa Fluor 488-conjugated secondary antibodies (1:500; Cat #A-21206; Invitrogen, Carlsbad, CA, United States) and 300 nM DAPI (Invitrogen, Carlsbad, CA, United States). Cells were visualized by the EVOS M7000 cell imaging system (Invitrogen, Carlsbad, CA, United States), and DAPI^+^ and BrdU^+^ cells were counted by ImageJ software. Quantification of BrdU^+^ cells were performed from 10 random microscope fields, including >2,000 nuclei for each group.

### Cell cycle analysis

Cell cycle analysis was performed as previously described [24]. Briefly, HepG2 or JHH-4 cells were harvested, washed twice with phosphate-buffered saline (PBS), and then treated with 70% ice-cold ethanol overnight at −20°C. Fixed cells were centrifuged at 600 g for 2 min, washed twice with PBS, and resuspended in a dye mixture containing 100 μg/mL RNase A (Thermo Scientific, Carlsbad, CA, United States; Cat #EN0531) and 50 μg/mL Propidium Iodide (Thermo Scientific, Carlsbad, CA, United States; Cat #P1304MP) in PBS. After overnight staining at 4°C, cells were analyzed for DNA content by the MACSQuant® X Flow Cytometer (Miltenyi Biotec, Gaithersburg, MD, USA). Flow cytometry data were analyzed and processed using FlowJo software (ver. 10.4, BD Biosciences Systems, San Diego, CA, USA) for differentiation of cells into G0/G1, S, and G2/M phases.

### Analysis of mRNA and miRNA expression

RNA isolation, reverse transcription, and qRT-PCR analysis of mRNA and miRNA were performed as previously described [23]. Data were analyzed using the 2^−ΔΔCt^ method and normalized to expression of RPL19 and U6 for analysis of mRNA and miRNA, respectively. All primers used for qRT-PCR can be found in Suppl. Table 1.

### Western blot analysis

**Supplemental Figure 1:**
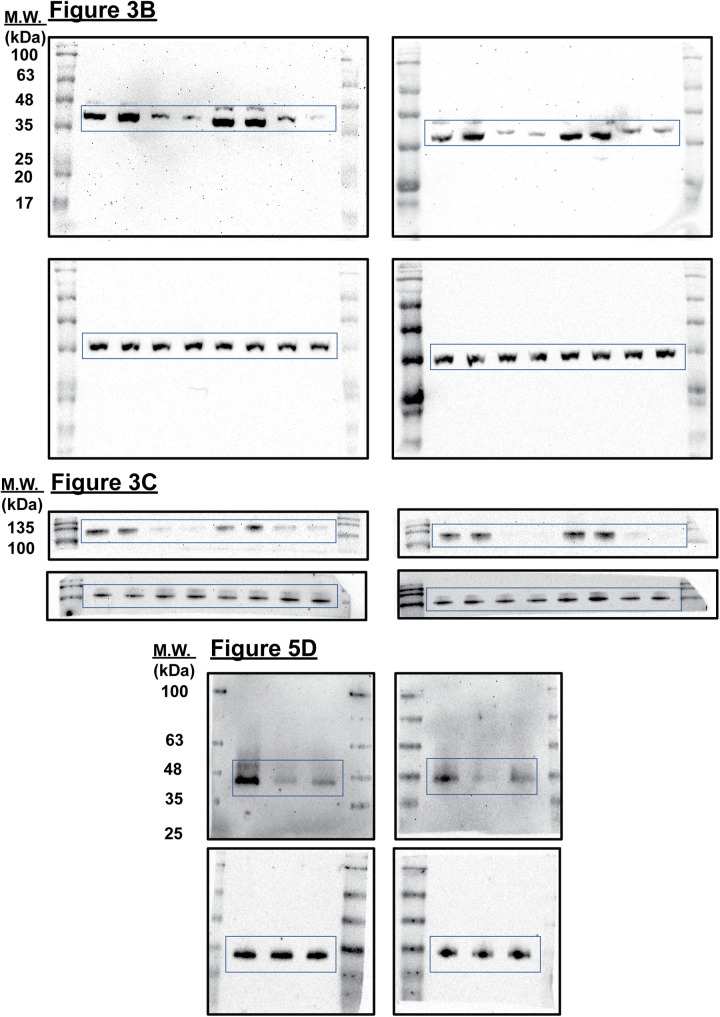
Uncropped immunoblots presented in this study.

Protein lysates were prepared in RIPA lysis buffer, separated by 12% SDS-PAGE gel, and transferred to a nitrocellulose membrane (Cytiva, Marlborough, MA, United States) using a semi-dry method. Membranes were then probed overnight with the following primary antibodies against CDK6 (1:1,000; Santa Cruz; Cat #sc-7961), Cyclin A2 (CST, Danvers, MA, United States; Cat #4656T), PLK1 (CST; Cat #4513T), GAPDH (1:2,500; Invitrogen; Cat #437000), total Rb (1:500; Santa Cruz; Cat #sc-102), and phosphorylated Rb (1:1,000; CST; Cat #8516T for Ser807/811, #9307T for Ser780, and #9301T for Ser795). HRP-conjugated goat anti-mouse IgG and goat anti-rabbit IgG (1:5,000; CST; Cat #7076S and #7074, respectively) were used as secondary antibodies. Blots were imaged using a UVP ChemStudio instrument (Analytik Jena, Beverly, MA, United States). Uncropped blots were shown in Suppl. Fig. 1.

### Prediction of miRNA targets and dual luciferase reporter assay

Putative miRNA targets of H19 were obtained from LncBase (www.microrna.gr/LncBase) and LncBook (https://ngdc.cncb.ac.cn/lncbook) databases [25,26]. The hybridization pattern between miRNA and the 3′ untranslated region (3′ UTR) of *CDK6* was predicted by using the DIANA-microT web server (http://www.microrna.gr/webServer) and the RNA hybrid tool (http://bibiserv.techfak.uni-bielefeld.de/rnahybrid/) [27,28]. Wild-type and mutant sequences of H19 and *CDK6* 3′ UTR were cloned into pmirGLO plasmid, and the miR-107 sequence was cloned into pSilencer plasmid as previously described [23]. Primers used for the cloning can be found in Suppl. Table 1. The interaction between miR-107 and its putative binding sites was tested by the Dual-Luciferase® Reporter Assay System (Promega, Madison, WI, United States) following the manufacturer’s instruction. Briefly, HEK293T cells were seeded in a 96-well plate and transfected with 300 ng of the luciferase reporter constructs (pmirGLO plasmid) and 300 ng of the pSilencer-miR-107 plasmid using Lipofectamine® 3000 Transfection Reagent (Invitrogen, Carlsbad, CA, United States) according to the manufacturer’s protocol. After 48 h, cells were lysed, incubated with firefly and Renilla luciferase substrates, and measured for enzyme activity using a luminometer. Firefly luciferase activity is normalized against Renilla luciferase to represent the miRNA binding efficiency.

### Statistical analysis

Data are presented as the mean ± standard deviation of the mean (SD) and statistically analyzed by the GraphPad Prism software version 9.5.1 (GraphPad) using unpaired Student’s *t*-test or one-way ANOVA. Three or more replicates from at least two independent experiments were used for the determination of significant differences. A *p* value of <0.05 was considered statistically significant.

## Results

### Manipulation of H19 levels in HCC cells affect proliferation, the cell cycle, and apoptosis

**FIGURE 1:**
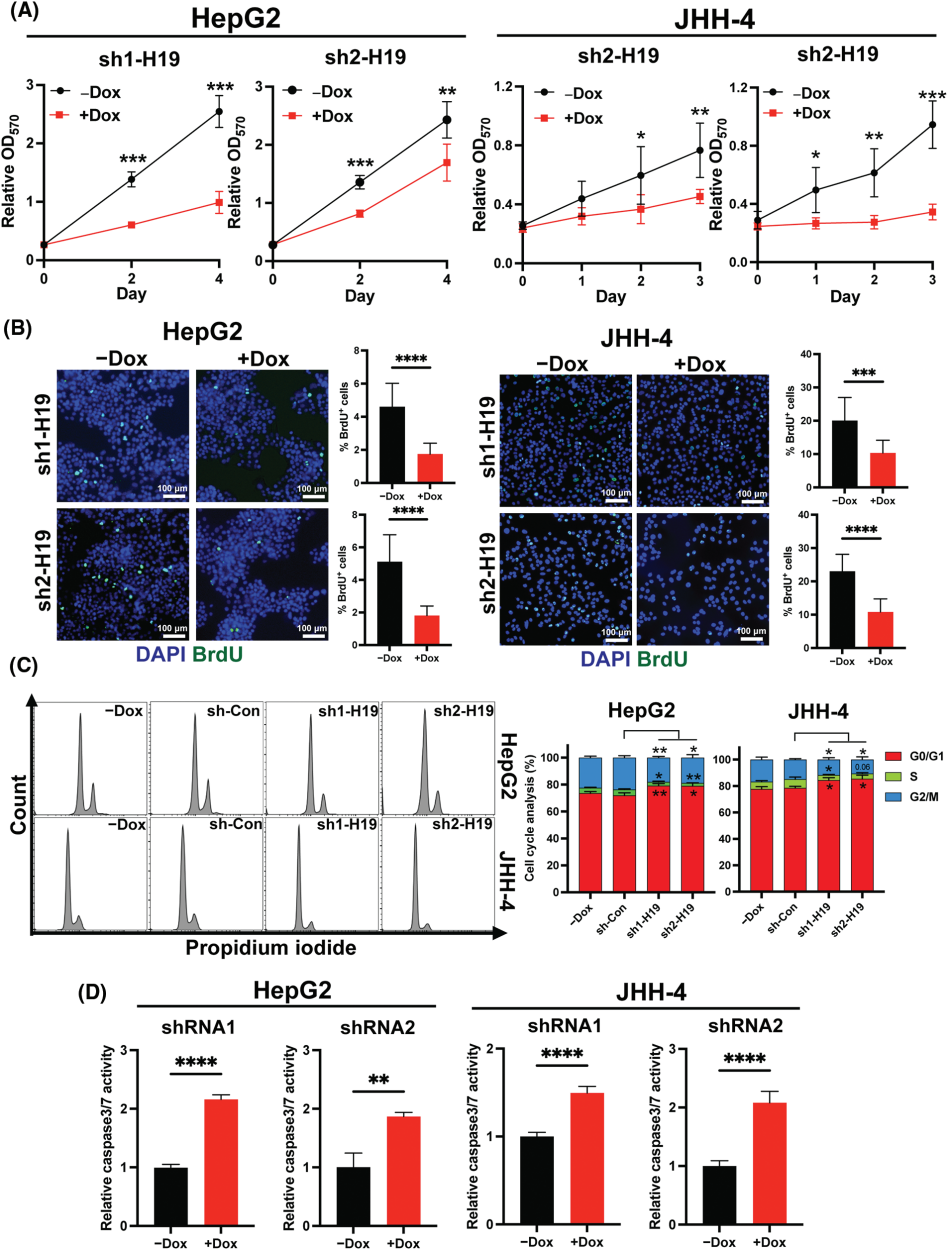
H19 depletion impairs cell growth, the cell cycle, and BrdU incorporation while promoting apoptosis in HCC cells. (A) MTT assay of HCC cells (HepG2 and JHH-4) with shRNA-mediated knockdown of H19 (sh-H19) induced by the presence of doxycycline (Dox). (B) BrdU incorporation assay of HCC cells with or without Dox treatment. (C) Cell cycle analysis of HepG2 and JHH-4 cells with -Dox, sh-control (sh-Con), and sh-H19 treatment. (D) Detection of apoptosis by levels of caspase3/7 activity in HCC cells with or without Dox treatment. Data are presented as the mean ± SD. N > 3 replicates per group from at least two independent experiments. Student’s *t*-test; *p < 0.05, **p < 0.01, ***p < 0.001 and ****p < 0.0001. Fluorescence images taken at equivalent exposures for comparison. Scale bars, 100 μm. DAPI: 4′,6-diamidino-2-phenylindole.

**FIGURE 2:**
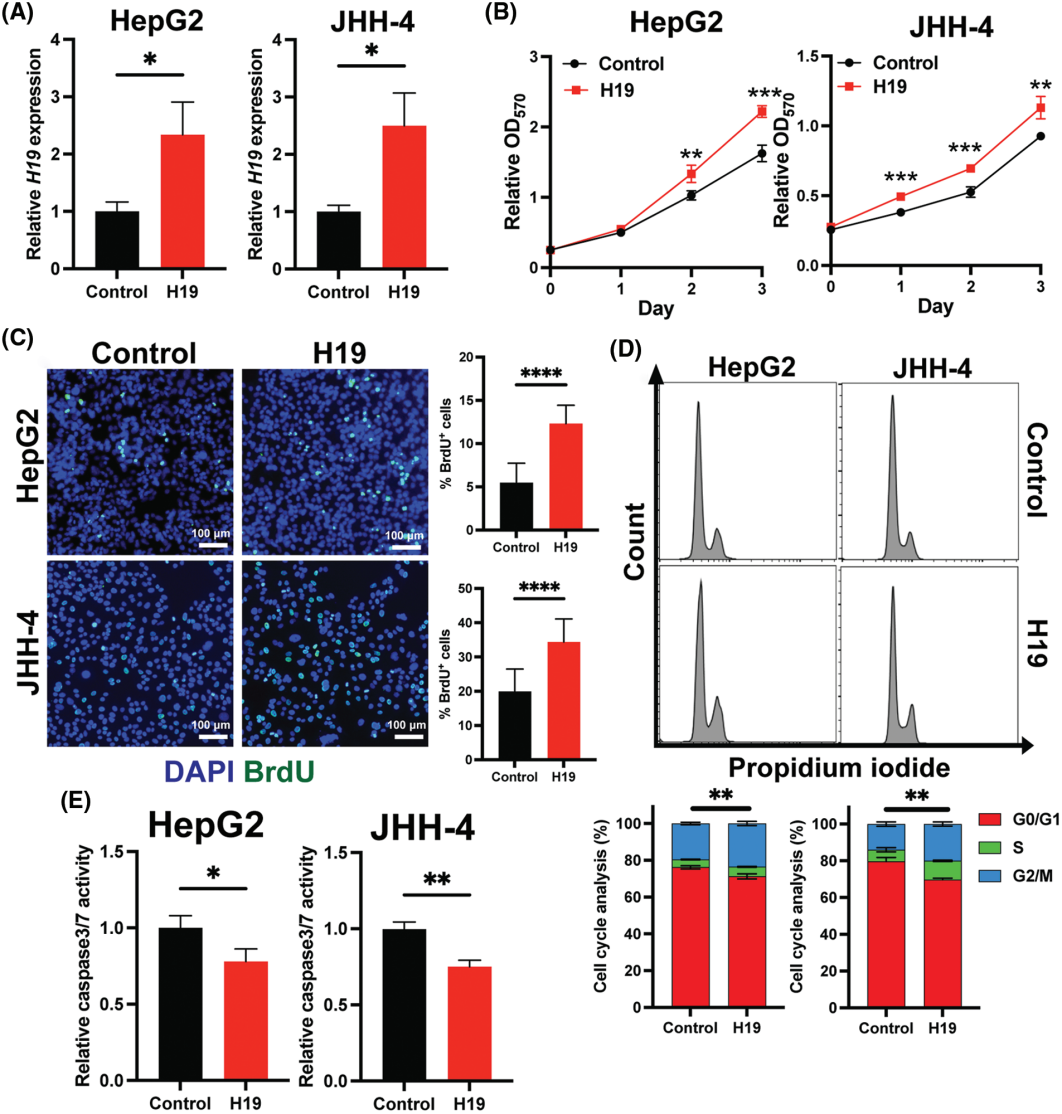
Overexpression of H19 promotes cell growth, the cell cycle, and BrdU incorporation while suppressing apoptosis in HCC cells. (A) qRT-PCR analysis of *H19* transcripts from HCC cells transfected with a control or H19-overexpressing plasmid. (B) MTT assay of HCC cells (HepG2 and JHH-4) with a control or H19-overexpressing plasmid. (C) BrdU incorporation assay of HCC cells with or without H19 overexpression. (D) Cell cycle analysis of HCC cells with or without H19 overexpression. (E) Detection of apoptosis by levels of caspase3/7 activity in HCC cells with or without H19 overexpression. Data are presented as the mean ± SD and expressed relative to those of the control (set as 1.0) for qRT-PCR experiments. N > 3 replicates per group from at least two independent experiments. Student’s *t*-test; *p < 0.05, **p < 0.01, ***p < 0.001 and ****p < 0.0001. Fluorescence images taken at equivalent exposures for comparison. Scale bars, 100 μm. DAPI: 4′,6-diamidino-2-phenylindole.

**Supplemental Figure 2:**
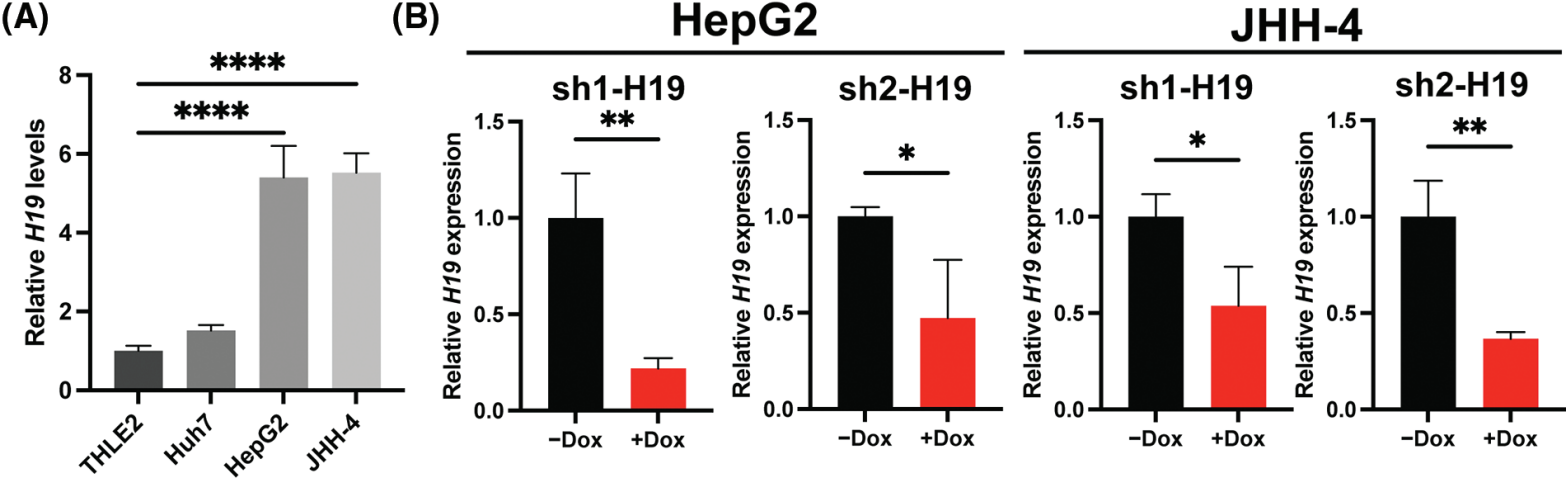
H19 expression and doxycycline (Dox)-inducible system of H19 knockdown in HCC cell lines. (A) qRT-PCR analysis of *H19* transcripts in normal hepatocytes (THLE-2) and HCC cell lines. (B) qRT-PCR analysis of *H19* transcripts upon shRNA-mediated knockdown of H19 (sh-H19) induced by the presence of Dox. Data are presented as the mean ± SD and expressed relative to those of THLE-2 or -Dox (set as 1.0) for qRT-PCR experiments. N > 3 replicates per group from at least two independent experiments. Student’s *t*-test or one-way ANOVA; *p < 0.05, **p < 0.01, ***p < 0.001 and ****p < 0.0001.

**Supplemental Figure 3:**
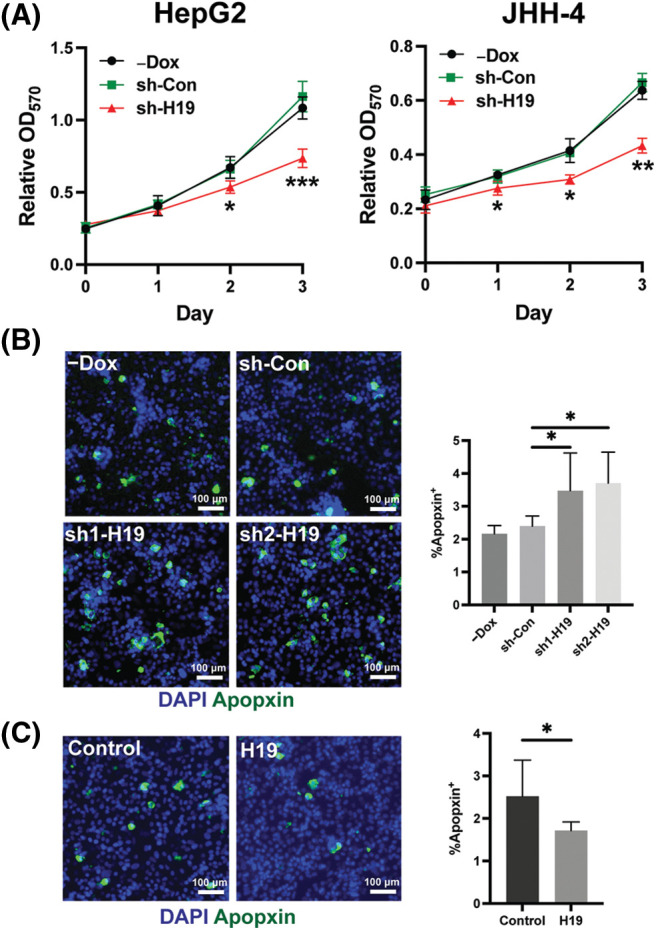
Role of H19 in the proliferation and apoptosis of HCC cells. (A) MTT assay of HCC cells with -Dox, shRNA-control (sh-Con), and shRNA-H19 (sh-H19) treatment. (B) Apopxin staining of HCC cells with -Dox, sh-Con, and sh-H19 treatment. (C) Apopxin staining of control and H19-overexpressing HCC cells. Data are presented as the mean ± SD. N > 3 replicates per group. A total of >10,000 nuclei were included for the quantification of Apopxin^+^ cells. Student’s *t*-test or one-way ANOVA; *p < 0.05, **p < 0.01, and ***p < 0.001. Fluorescence images taken at equivalent exposures for comparison. Scale bars, 100 μm. DAPI: 4′,6-diamidino-2-phenylindole.

To explore the role of H19 in cell growth, we determined the effect of knockdown of H19 on the proliferation of HCC cells. First, we confirmed the upregulation of H19 in HCC cells in comparison with a normal hepatocyte cell line, consistent with previous clinical data where H19 is found upregulated in HCC tissues (Suppl. Fig. 2A) [10–12]. Then, we established a doxycycline (Dox)-inducible H19 knockdown system in two independent HCC cell lines (HepG2 and JHH-4). Upon Dox treatment, levels of *H19* transcripts were significantly downregulated (Suppl. Fig. 2B). To determine cell growth, we performed the MTT assay and found that H19 suppression impaired cell proliferation (Fig. 1A and Suppl. Fig. 3A). Consistent with the results of the MTT assay, we observed a significantly reduced number of BrdU-positive (BrdU^+^) cells upon downregulation of H19 (Fig. 1B). Cell cycle analysis revealed that H19 knockdown significantly decreased the distribution of cells in S and G2/M phases while increasing the number of cells arrested in the G1 phase (Fig. 1C). Moreover, apoptosis detection by caspase-3/7 activation and Apopxin staining was significantly elevated in H19-knockdown cells (Fig. 1D and Suppl. Fig. 3B). Conversely, overexpression of H19 promoted cell growth, BrdU incorporation, and cell cycle progression while reducing apoptosis in HCC cells (Figs. 2A–2E and Suppl. Fig. 3C). Taken together, these results suggest the oncogenic role of H19 in HCC, and its upregulation may be crucial to drive the proliferation and the cell cycle of HCC cells.

### Downregulation of H19 inhibits the expression of CDK6

**Figure 3:**
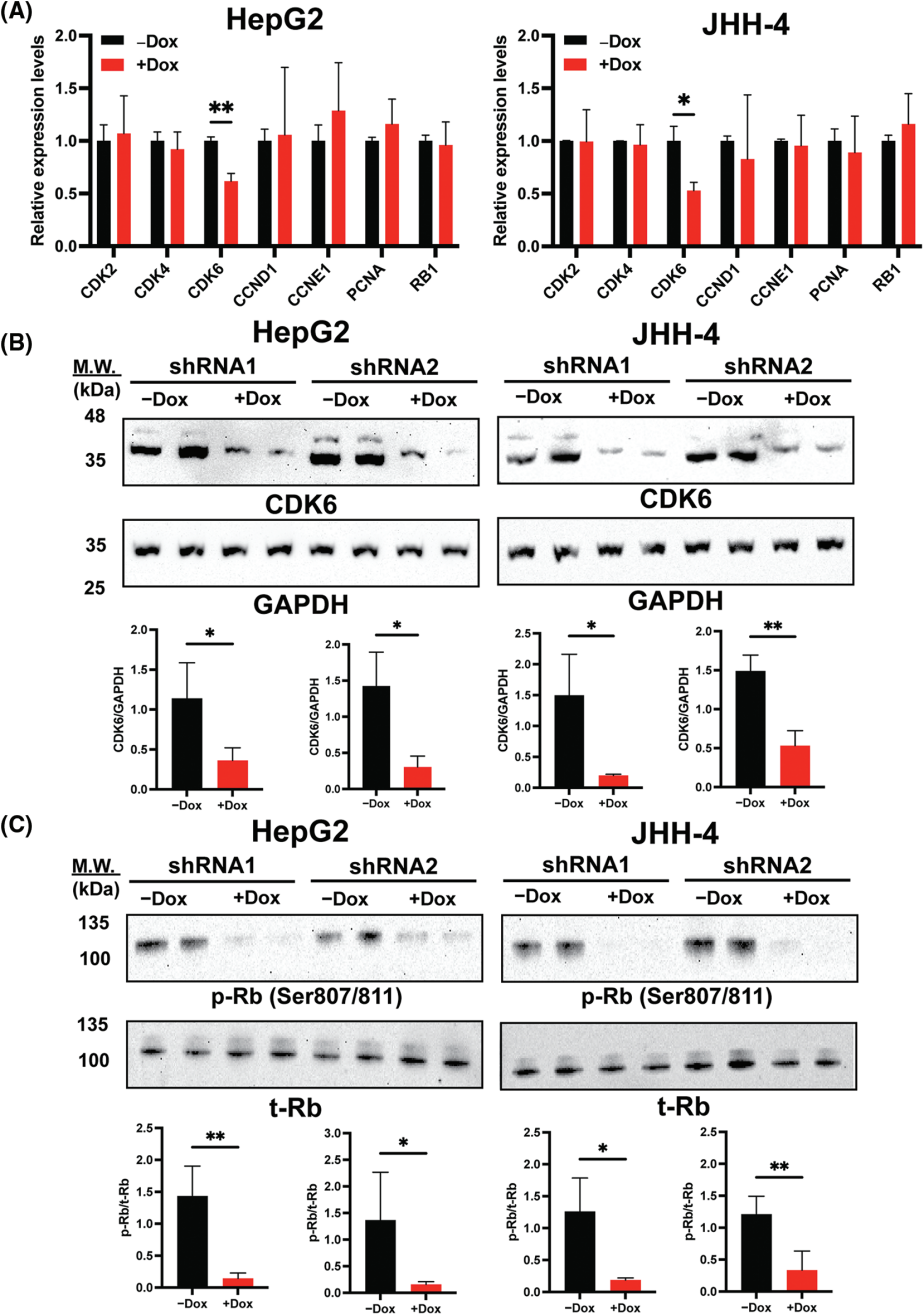
H19 suppression downregulates the expression of CDK6 in HCC cells. (A) qRT-PCR analysis of key cell cycle genes in HCC cells upon shRNA-mediated depletion of H19 induced by the presence of doxycycline (Dox). (B) Western blot of CDK6 in HCC cells (HepG2 and JHH-4) with or without Dox treatment. GAPDH was used as a loading control. (C) Western blot of phosphorylated Rb (p-Rb) and total Rb (t-Rb) in HCC cells with or without Dox treatment. Data are presented as the mean ± SD and expressed relative to those of-Dox (set as 1.0) for qRT-PCR experiments. N = 3 replicates per group from at least two independent experiments. Student’s *t*-test; *p < 0.05 and **p < 0.01.

**Supplemental Figure 4:**
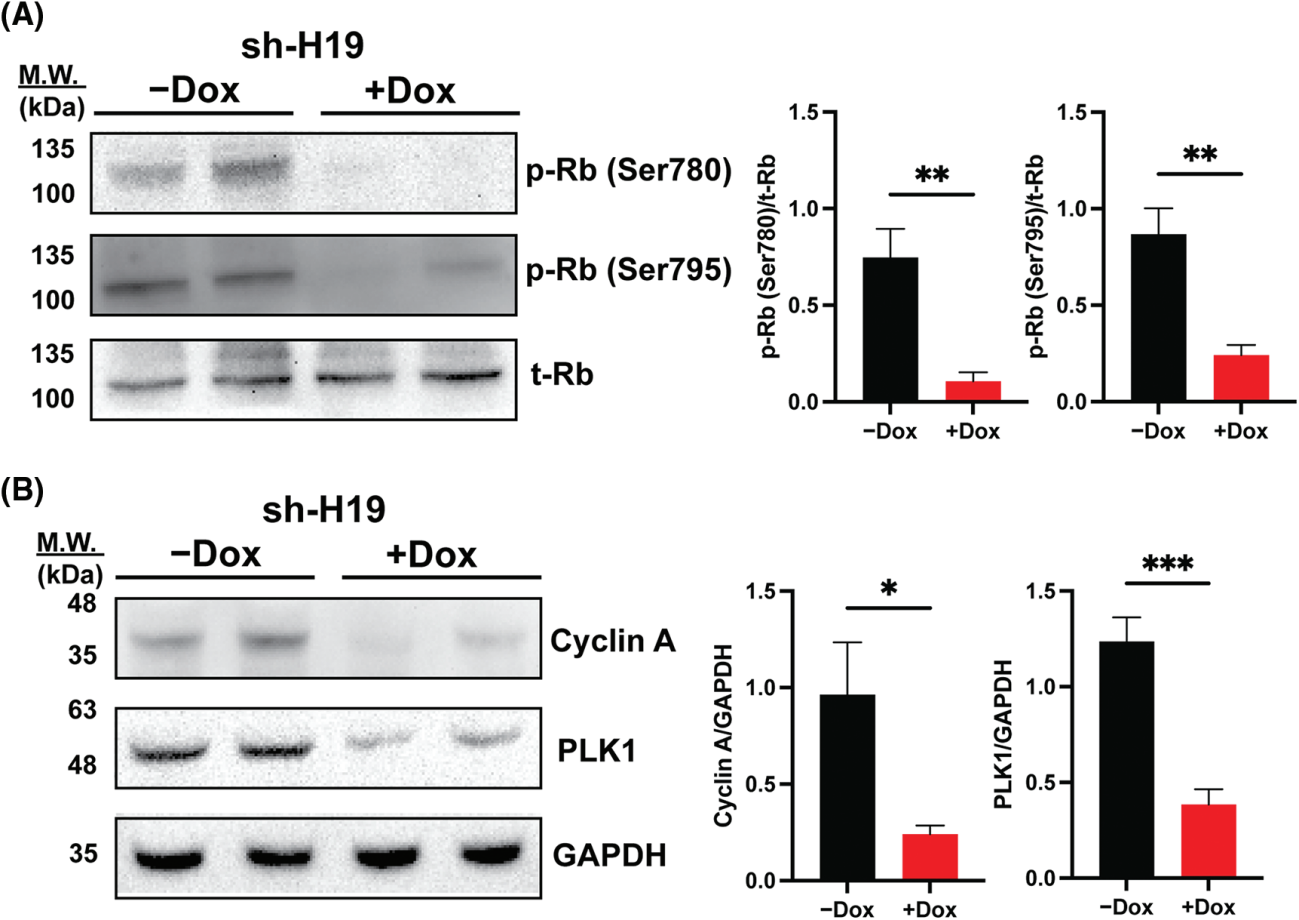
Rb phosphorylation and expression of E2F target genes upon shRNA-mediated knockdown of H19 (sh-H19) induced by doxycycline (Dox). (A) Western blot of phosphorylated Rb at the Ser780 and Ser795 residues upon H19 knockdown. (B) Western blot analysis of Cyclin A2 and PLK1 upon H19 knockdown. Data are presented as the mean ± SD. N = 3 replicates per group. Student’s *t*-test; *p < 0.05, **p < 0.01, and ***p < 0.001.

As BrdU incorporation and cell cycle progression were impaired in H19-knockdown cells (Figs. 1B and 1C), we hypothesized that suppression of H19 could induce G1 phase arrest in the cell cycle. Thus, we surveyed the expression of key cell cycle genes that promote the S-phase entry by qRT-PCR analysis. We found that upon H19 depletion, levels of *CDK6* transcripts were significantly decreased in two independent HCC cell lines (Fig. 3A). Downregulation of CDK6 in H19-knockdown cells was further confirmed at the protein level by Western blot (Fig. 3B). CDK6 is a critical mediator in the cellular transition into the S phase, and its overexpression plays an important role in driving tumorigenesis in many cancer types [29,30]. CDK6 forms a protein complex with CDK4 and D-type cyclins to drive cell proliferation. A well-known substrate of this protein complex is the retinoblastoma protein (Rb), which inhibits cell cycle progression into the S phase until its inactivation by phosphorylation. Upon H19 knockdown expression, we observed a significant reduction in Rb phosphorylation at the Ser780, Ser795, and Ser807/811 residues, supporting the impaired function of CDK6 (Fig. 3C and Suppl. Fig. 4A). Rb phosphorylation weakens its association with the E2F transcription factor, allowing for activation of E2F-mediated transcription and the S-phase entry [31]. Accordingly, we observed downregulation of E2F target genes such as Cyclin A2 and PLK1 in H19-knockdown cells (Suppl. Fig. 4B). Together, these data indicate that H19 depletion could impair the proliferation of HCC cells at least by suppressing the activity of CDK6.

### MicroRNA-107 directly interacts with H19 and 3′ UTR of CDK6 transcripts

**FIGURE 4:**
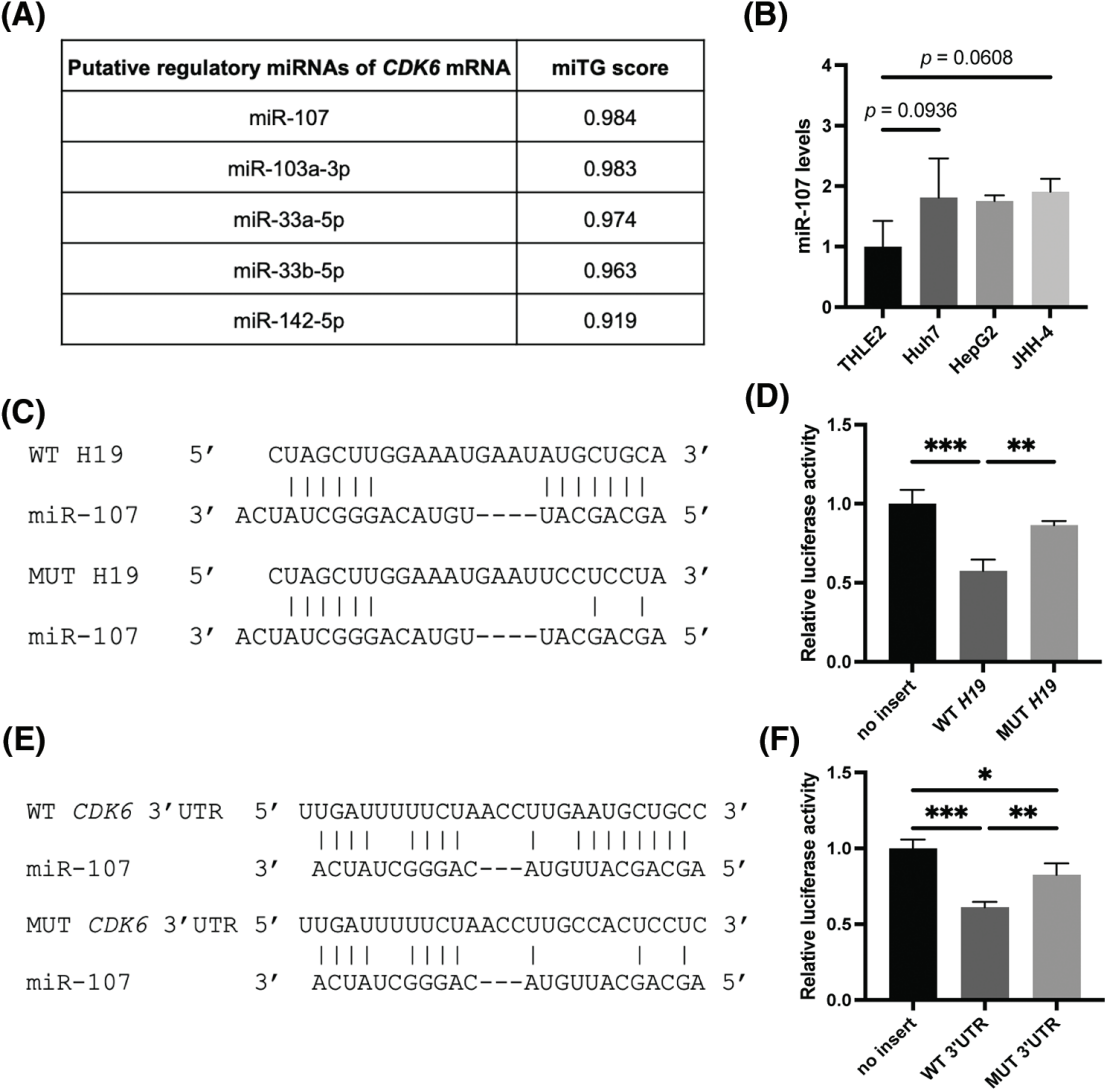
MiR-107 directly interacts with H19 and the 3′ UTR of CDK6 mRNA. (A) Ranking of H19-associated miRNAs that are predicted to interact with the 3′ UTR of CDK6 mRNA based on miRNA target gene (miTG) scores. (B) qRT-PCR analysis of miR-107 in normal hepatocytes (THLE-2) and HCC cell lines. (C) Hybridization pattern of wild-type (WT) and mutant (MUT) H19 sequences with miR-107. (D) Dual luciferase reporter assay of wild-type (WT) and mutant (MUT) H19 sequences with miR-107. (E) Hybridization pattern of wild-type (WT) and mutant (MUT) CDK6 3′ UTR sequences with miR-107. (F) Dual luciferase reporter assay of wild-type (WT) and mutant (MUT) CDK6 3′ UTR sequences with miR-107. Data are presented as the mean ± SD and expressed relative to those of THLE-2 (set as 1.0) for qRT-PCR experiments. N = 3 replicates per group from at least two independent experiments. One-way ANOVA; *p < 0.05, **p < 0.01 and ***p < 0.001.

LncRNAs can exert their regulatory function by acting as sponges of microRNAs (miRNAs), which control gene expression post-transcriptionally by binding target mRNAs at the 3’ UTR, resulting in mRNA degradation and/or translational repression [32]. Thus, lncRNAs can inhibit the activity of miRNAs and allow upregulation of miRNA target genes. Given this mode of action, we hypothesized that H19 could sequester a miRNA that negatively regulates the expression of CDK6. To identify such miRNAs, we obtained two lists of miRNAs that were previously reported or predicted to interact with H19 from the lncBase and lncBook databases [25,26]. A total of 54 miRNAs were found to overlap from the two sources (Suppl. Table 2). Then, we employed the DIANA-microT web server to rank the interaction of these miRNAs with the sequence of *CDK6* 3’UTR and found that miR-107 achieved the highest miRNA target gene (miTG) score (Fig. 4A) [27]. Using the RNA hybrid tool, we analyzed the sequence of H19 and revealed multiple binding sites of miR-107 with minimal free energy (MFE) <−20 kcal/mol (Suppl. Table 3) [28]. This bioinformatic analysis indicated that H19 could serve as a molecular sponge for miR-107 and hence promote the expression of CDK6. Supporting this notion, we observed that levels of miR-107 were slightly increased, although not significantly, in HCC cells, and overexpression of H19 could be a way to counter the antiproliferative activity of miR-107 (Suppl. Fig. 1A and Fig. 4B).

To test whether miR-107 was sponged by H19, we performed a dual luciferase reporter gene assay in HEK293 cells, a common model used for testing miRNA-target interactions [33,34]. Results showed that miR-107 significantly reduced the luciferase activity of the H19 wild-type (WT) reporter, while this inhibition was alleviated with the H19 mutant (MUT) sequence (Figs. 4C and 4D). To test if CDK6 is a direct target of miR-107, we performed another set of dual luciferase reporter gene assay and demonstrated that miR-107 directly interacted with the 3′ UTR of *CDK6* mRNA (Figs. 4E and 4F). Collectively, we illustrated that H19 could sequester miR-107 to promote CDK6 expression.

### Inhibition of miR-107 partially rescues a proliferative defect induced by H19 depletion

**Figure 5:**
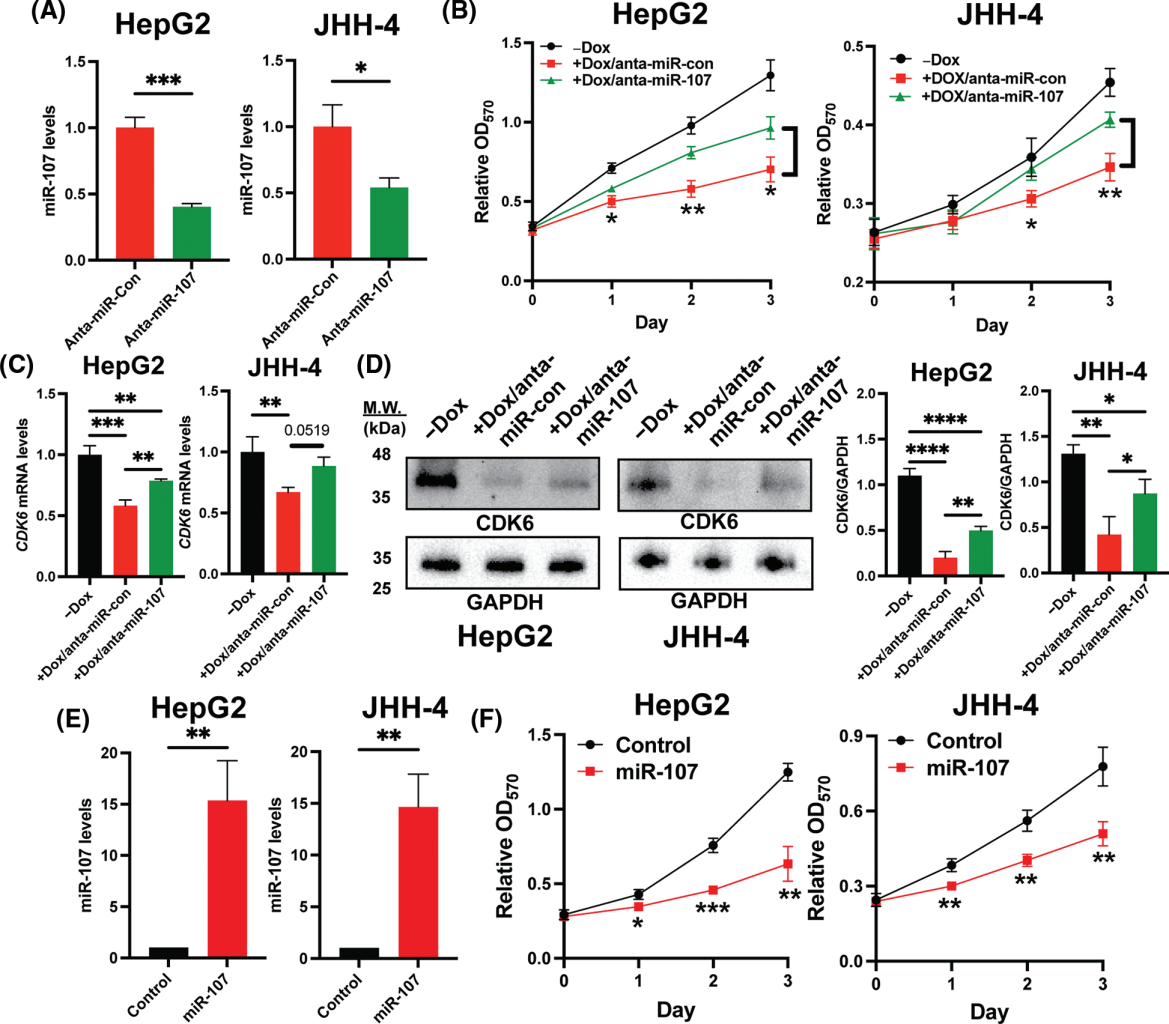
Inhibition of miR-107 alleviated growth suppression by H19 silencing in HCC cells. (A) qRT-PCR analysis of miR-107 in HCC cells treated with control antagomir (anta-miR-con) and miR-107 antagomir (anta-miR-107). (B) MTT assay of H19-depleted HCC cells (HepG2 and JHH-4) treated with anta-miR-con and anta-miR-107. (C) qRT-PCR analysis of CDK6 mRNA in H19-depleted HCC cells treated with anta-miR-con and anta-miR-107. (D) Western blot of CDK6 in H19-depleted HCC cells treated with anta-miR-con and anta-miR-107. (E) qRT-PCR analysis of miR-107 after its overexpression in HCC cells. (F) MTT assay of control and miR-107-overexpressing HCC cells. Data are presented as mean ± SD and expressed relative to those of anta-miR-con, -Dox, or Control (set as 1.0) for qRT-PCR experiments. N = 3 replicates per group from at least two independent experiments. Student’s *t*-test or one-way ANOVA; *p < 0.05, **p < 0.01 and ***p < 0.001.

To confirm that miR-107 is crucial in the regulation of the H19/miR-107/CDK6 axis, we introduced control antagomir (anta-miR-con) or miR-107 antagomir (anta-miR-107) into H19-knockdown HCC cells. We first showed that anta-miR-107 efficiently blocked the activity of miR-107 (Fig. 5A). Then, we tested the proliferation of HCC cells with antagomirs by the MTT assay, and the results suggest that inhibition of miR-107 partially attenuated the antiproliferative effect mediated by H19 depletion (Fig. 5B). Consistently, expression of CDK6 was significantly upregulated at both mRNA and protein levels upon miR-107 inhibition in H19-knockdown cells (Figs. 5C and 5D). To confirm the antiproliferative role of miR-107, we overexpressed miR-107 in HepG2 and JHH-4 cells and observed growth suppression (Figs. 5E and 5F). Taken together, these results indicate that H19 could play an important role in sequestering miR-107 and promoting CDK6 expression, thus driving proliferation in HCC cells.

## Discussion

**Figure 6:**
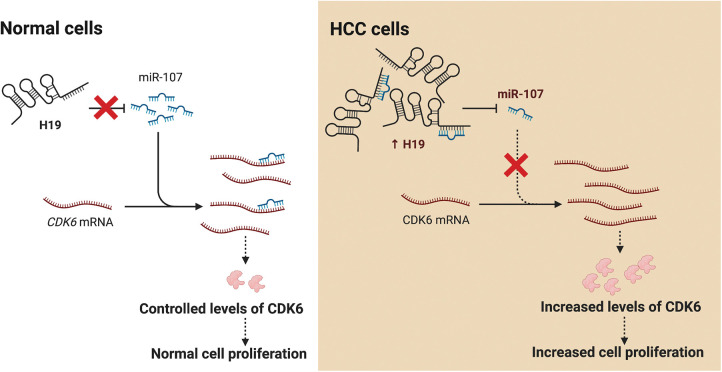
H19 promotes the proliferation of HCC cells via the miR-107/CDK6 axis. In normal cells, miR-107 posttranscriptionally regulates levels of CDK6 and drives controlled cell division. In HCC cells, upregulation of H19 sequesters miR-107 and allows overexpression of CDK6, which leads to uncontrolled cell proliferation.

LncRNA H19 has been widely investigated as a biomarker and therapeutic target in various types of cancers including HCC. Nevertheless, mechanistic insights into the role of H19 in cell cycle regulation are still lacking. In this study, we showed that H19 suppression in HCC cells reduced their proliferation and impaired the G1-to-S transition in the cell cycle. Mechanistically, H19 could sponge miR-107 and inhibit its function to downregulate the expression of CDK6, which is a key mediator in driving cell cycle progression. Thus, H19 depletion allows miR-107 to suppress CDK6 expression and arrest cells in the G1 phase. Collectively, we proposed the H19/miR-107/CDK6 axis as a novel regulatory mechanism to promote proliferation in HCC cells (Fig. 6).

Although the role of H19 in HCC have been controversial, most studies, including ours, suggest its oncogenic function in promoting the initiation and progression of HCC [16,17,35]. In addition, we identified an anti-cancer role of miR-107 in HCC as a regulator of CDK6 in the cell cycle. Supporting this observation, previous studies have shown that miR-107 directly targets CDK6 and induces the cell cycle G1 arrest in gastric, bladder, and non-small cell lung cancer cells [36–38]. Furthermore, the tumor suppressing function of miR-107 has been previously reported in HCC via targeting oncogenic genes, including RGS4, HMGA2, HMGCS2, cofilin-1, and Wnt/β-catenin [39–43]. MiR-107 can also be used as a circulating and tissue biomarker for early detection of HCC [44,45]. Conversely, a few studies show that miR-107 accelerates the tumor progression via amplification of EGFR and Wnt signaling [46,47]. The conflicting effects of miR-107 on tumor cell regulation have been reported in other cancers [48]. Therefore, further investigation of miR-107 is warranted to resolve its controversial function in HCC.

CDK6 is a long-known critical mediator that promotes the G1 to S transition in the cell cycle; hence, its overexpression is unsurprisingly reported in many types of cancer cells to drive cell division [29]. To interrupt this process, CDK inhibitors have been developed for cancer treatment with the success of FDA-approved CDK4/6 inhibitors to treat certain types of hormone receptor-positive, HER2-negative breast cancers [30]. However, resistance to CDK4/6 inhibitors is prevalent in other cancer cell types including HCC, thus limiting the utility of these drugs in clinics [49]. A recent study suggests that CDK6-deficient cancer cells that rely on the function of CDK4 in cell cycle progression are sensitive to these inhibitors; in contrast, upregulation of CDK6 in breast cancer cells promotes CDK4/6 inhibitor resistance [50–52]. As our data showed that H19 depletion downregulated the expression of CDK6, silencing H19 could desensitize HCC cells to CDK4/6 inhibitors. Future experiments should be carried out to determine the combination of H19 silencing and CDK4/6 inhibitors as a novel synergistic regimen for HCC treatment.

In summary, we present a novel function of H19 in the regulation of miR-107 and CDK6 to promote the proliferation of HCC cells. H19 promotes CDK6 overexpression by acting as a sponge for miR-107, which directly targets CDK6 expression. A similar mechanistic mode has been proposed in bladder carcinoma where circular RNA TCF25 serves as a molecular sponge for miR-107 to upregulate CDK6 expression and promote proliferation [37]. Alternatively, H19 could drive cell growth in glioma cells by sequestering miR-200a, which negatively regulates CDK6 expression [53]. Together, the H19/miR-107/CDK6 axis could be exploited as a new target for both diagnosis and treatment of HCC and other cancers.

## Supplementary Materials

**Supplemental Table 1:** Primer sequences used in this study

	Cloning primers
Sense_sh1_H19	ccggGACGTGACAAGCAGGACATGActcgagTCATGTCCTGCTTGTCACGTCttttt
Antisense_sh1_H19	aattaaaaaGACGTGACAAGCAGGACATGActcgagTCATGTCCTGCTTGTCACGTC
Sense_sh2_H19	ccggGCACTACCTGACTCAGGAATCctcgagGATTCCTGAGTCAGGTAGTGCttttt
Antisense_sh2_H19	aattaaaaaGCACTACCTGACTCAGGAATCctcgagGATTCCTGAGTCAGGTAGTGC
Sense_sh_control	ccggCCTAAGGTTAAGTCGCCCTCGctcgagCGAGGGCGACTTAACCTTAGGttttt
Antisense_sh_control	aattaaaaaCCTAAGGTTAAGTCGCCCTCGctcgagCGAGGGCGACTTAACCTTAGG
Sense_miR-107	ccggAGCAGCATTGTACAGGGCTATCActcgagTGATAGCCCTGTACAATGCTGCTttttt
Antisense_miR-107	aattaaaaaAGCAGCATTGTACAGGGCTATCActcgagTGATAGCCCTGTACAATGCTGCT
Sense_wt_H19	CGCGGCCGCtctagcttggaaatgaatatgctgcacC
Antisense_wt_H19	TCGAGgtgcagcatattcatttccaagctagaGCGGCCGCGAGCT
Sense_mut_H19	CGCGGCCGCtctagcttggaaatgaatTCCTCCTacC
Antisense_mut_H19	TCGAGgtAGGAGGAattcatttccaagctagaGCGGCCGCGAGCT
Sense_wt_CDK6_ 3′ UTR	CGCGGCCGCtttgatttttctaaccttgAATGCTGCcaC
Antisense_wt_CDK6 _3′ UTR	TCGAGtgGCAGCATTcaaggttagaaaaatcaaaGCGGCCGCGAGCT
Sense_mut_CDK6_ 3′ UTR	CGCGGCCGCtttgatttttctaaccttgCCACTCCTcaC
Antisense_mut_H19_ CDK6_3′ UTR	TCGAGtgAGGAGTGGcaaggttagaaaaatcaaaGCGGCCGCGAGCT
F_miR-107_cloning	gctGAATTCaagaaggcactggatgataatg
R_miR-107_cloning	cgaGGATCCgcctcaactcctctttcctg

qRT-PCR primers	
F_H19	CACTGGCCTCCAGAGCCCGT
R_H19	CGTCTTGGCCTTCGGCAGCTG
F_RPL19	GCTCTTTCCTTTCGCTGCT
R_RPL19	CATTGGTCTCATTGGGGTCT
F_U6	CTCGCTTCGGCAGCACA
F_miR-107	AGCATTGTACAGGGCTATGA
R_universal_miR	GCAGGGTCCGAGGTATTC
F_CDK2	GCATCTTTGCTGAGATGGTGACT
R_CDK2	TCATCCAGGGGAGGTACAACTT
F_CDK4	TGTATGGGGCCGTAGGAAC
R_CDK4	CTCAGATCAAGGGAGACCCTCAC
F_CDK6	GAGAGCCGACTGACACTC
R_CDK6	CAGAATCATTGCACCTGAGG
F_CCND1	GACCCCGCACGATTTCATTG
R_CCND1	AATGAACTTCACATCTGTGGCAC
F_CCNE1	CCTGTACTGAGCTGGGCAAATA
R_CCNE1	ACTTCACACACCTCCATTAACCA
F_PCNA	GGCGTGAACCTCACCAGTATGT
R_PCNA	CCGCGTTATCTTCGGCCCTT
F_RB	GGTATGTAACAGCGACCGTG
R_RB	TCCTGGGAGATGTTTACTTCCATC

**Supplemental Table 2:** List of potential miRNAs associated with H19 based on the LncBase and LncBook databases

hsa-let-7i-3p	hsa-miR-132-5p	hsa-miR-17-5p	hsa-miR-191-3p	hsa-miR-20b-5p	hsa-miR-93-5p	hsa-miR-107	hsa-miR-103a-3p	hsa-miR-29b-3p	hsa-miR-29a-3p
hsa-miR-29c-3p	hsa-miR-141-3p	hsa-miR-200a-3p	hsa-miR-22-3p	hsa-miR-34a-5p	hsa-miR-193a-3p	hsa-miR-20a-5p	hsa-miR-106b-5p	hsa-miR-671-5p	hsa-miR-106a-5p
hsa-miR-627-5p	hsa-miR-194-5p	hsa-miR-141-5p	hsa-miR-93-3p	hsa-miR-342-3p	hsa-miR-138-5p	hsa-miR-152-3p	hsa-miR-148b-3p	hsa-miR-767-5p	hsa-miR-148a-3p
hsa-miR-196a-5p	hsa-miR-28-5p	hsa-miR-423-5p	hsa-miR-708-5p	hsa-miR-204-3p	hsa-miR-744-5p	hsa-miR-625-5p	hsa-miR-769-3p	hsa-miR-301b-3p	hsa-miR-301a-3p
hsa-miR-130b-3p	hsa-miR-130a-3p	hsa-miR-454-3p	hsa-miR-766-5p	hsa-miR-518c-5p	hsa-miR-769-5p	hsa-miR-129-2-3p	hsa-miR-19a-3p	hsa-miR-339-5p	hsa-miR-19b-3p
hsa-miR-18a-5p	hsa-miR-18b-5p	hsa-miR-675-5p	hsa-miR-24-1-5p						

**Supplemental Table 3:** Hybridization pattern between H19 and miR-107 with minimal free energy (MFE) <−20 kcal/mol as predicted by the RNA hybrid tool

Rank	Hybridization pattern	MFE (kcal/mol)
1	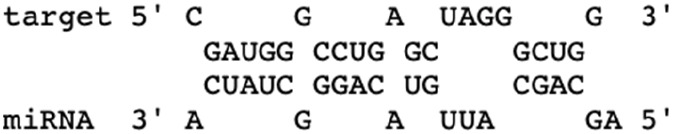	−27.6
2	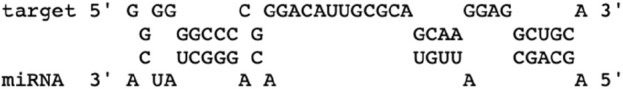	−25.8
3	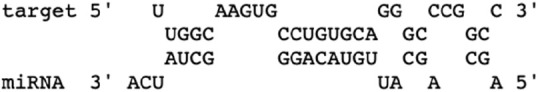	−25.7
4	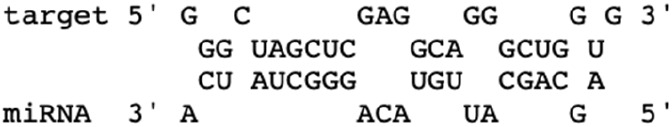	−25.1
5	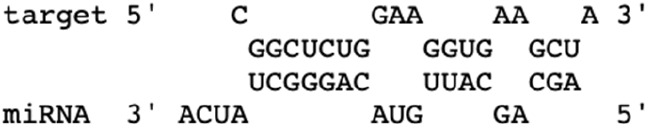	−24.5
6	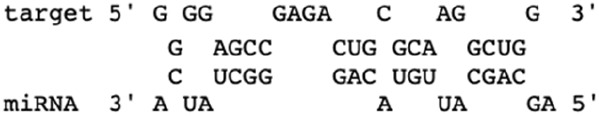	−24.2
7	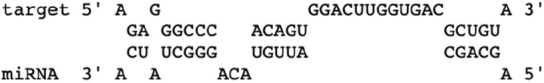	−23.8
8	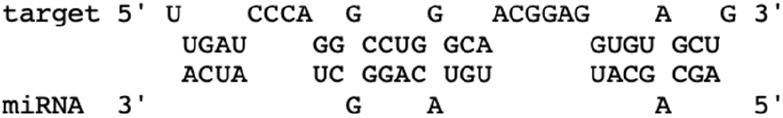	−23.7
9	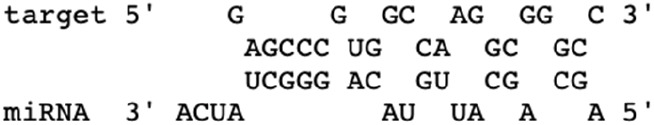	−23.7
10	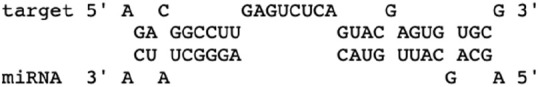	−23.3
11	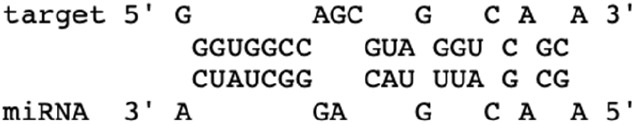	−23.0
12	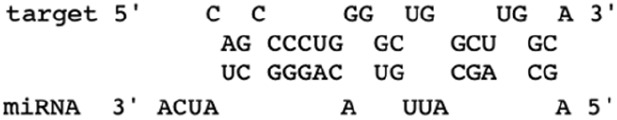	−22.5
13	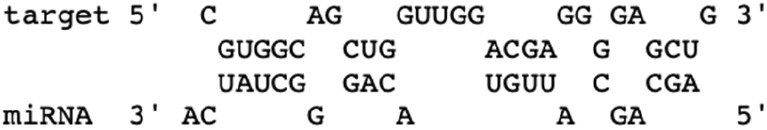	−22.5
14	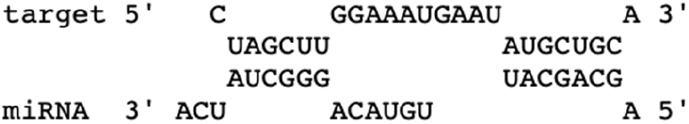	−22.4
15	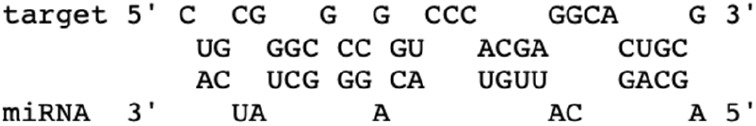	−22.3
16	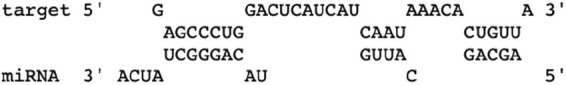	−22.3
17	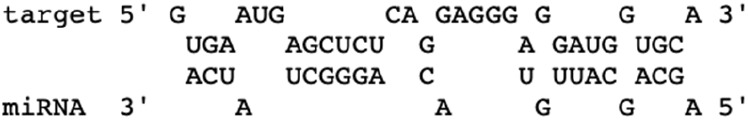	−22.1
18	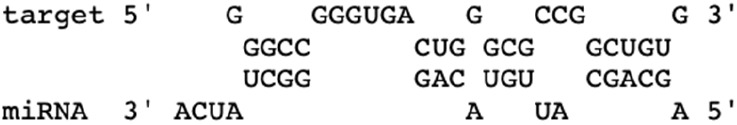	−21.9
19	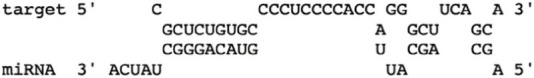	−21.5
20	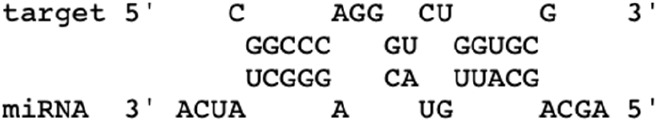	−21.4
21	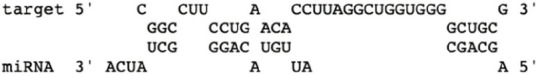	−21.2
22	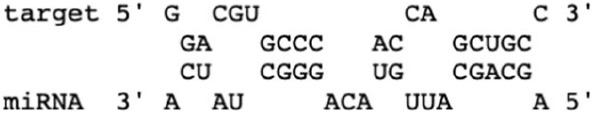	−20.9
23	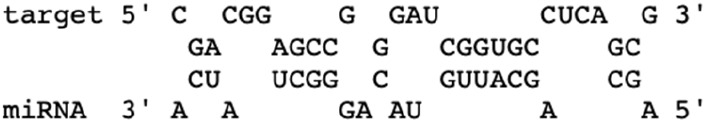	−20.9
24	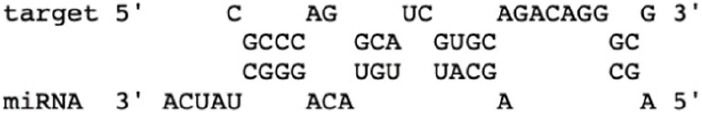	−20.8
25	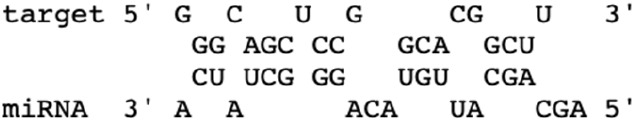	−20.8
26	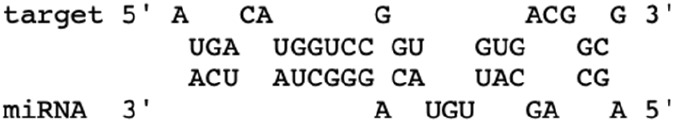	−20.4
27	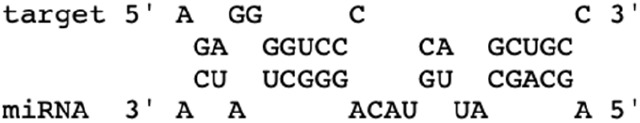	−20.4
28	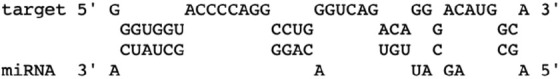	−20.0

## Data Availability

Raw data of all quantitatively analyzed experiments are available from the corresponding authors on reasonable request.
